# Subsequent Treatments After Progression on Cyclin-Dependent Kinase 4/6 Inhibitors: A Review of the Evidence and a Real-World Data Perspective From Portuguese Hospitals

**DOI:** 10.7759/cureus.109017

**Published:** 2026-05-17

**Authors:** Rita Freitas, Tiago Barroso, Sofia Braga, Catarina C Santos, Marta Batista, Andreia Chaves, Sara Cabral, Ana Fortuna, Vanessa Patel, João Araújo, Tânia Duarte, Tiago P Cabral, Sandra Silva, Cláudia Viana, Carlota Baptista, Joana Gonçalves, Inês Q Dunões, Inês Eiriz

**Affiliations:** 1 Department of Medical Oncology, Unidade Local de Saúde (ULS) de Amadora/Sintra, Lisbon, PRT; 2 Department of Medical Oncology, Unidade Local de Saúde (ULS) de Santa Maria, Lisbon, PRT; 3 Department of Medical Oncology, Hospital de Cascais, Cascais, PRT; 4 Department of Medical Oncology, Instituto Português de Oncologia de Lisboa Francisco Gentil, Lisbon, PRT; 5 Department of Medical Oncology, Unidade Local de Saúde (ULS) do Algarve, Faro, PRT; 6 Department of Medical Oncology, Unidade Local de Saúde (ULS) de Lisboa Ocidental, Lisbon, PRT; 7 Department of Medical Oncology, Unidade Local de Saúde (ULS) de Gaia/Espinho, Vila Nova de Gaia, PRT; 8 Department of Medical Oncology, Hospital do Divino Espírito Santo de Ponta Delgada, Ponta Delgada, PRT; 9 Department of Medical Oncology, Unidade Local de Saúde (ULS) de Loures/Odivelas, Loures, PRT; 10 Department of Medical Oncology, Unidade Local de Saúde (ULS) do Arco Ribeirinho, Barreiro, PRT; 11 Department of Medical Oncology, Unidade Local de Saúde (ULS) do Alentejo Central, Évora, PRT

**Keywords:** cyclin-dependent kinase 4/6 inhibitors, disease progression, endocrine therapy, metastatic breast cancer, treatment sequencing

## Abstract

Background: The treatment with cyclin-dependent kinase 4/6 inhibitors (CDK4/6i) (ribociclib, palbociclib, and abemaciclib) combined with endocrine therapy (ET) has become the standard of care (SoC) for first-line treatment in patients with metastatic hormone receptor-positive (HR+) and human epidermal growth factor receptor 2-negative (HER2-) breast cancer. There is a lack of consensus on what treatment to offer after disease progression, and there are no established guidelines for therapeutic sequencing.

Objective: This multicentric retrospective observational analysis aims to characterize systemic treatment following CDK4/6i, among patients with HR+/HER2- metastatic breast cancer (MBC).

Materials and methods: An observational retrospective study performed in 10 Portuguese oncological centers evaluated 237 patients with MBC who had been treated with at least one CDK4/6i, between January 2016 and July 2022, and had progressed with this treatment. We collected data on the initial staging, the date of diagnosis, HR status, Ki67 and HER2, the date of recurrence, metastatic site, the type of treatment, the date of initiation and the date of progression, and the reason for interruption.

Results: Rechallenging with a different CDK4/6 inhibitor demonstrated the highest overall survival (OS), though it is used in a small percentage of patients, highlighting a potential survival advantage and the need for further investigation into optimal sequencing strategies. While capecitabine showed favorable progression-free survival (PFS) and overall survival, ET alone provided a higher OS, reflecting the influence of disease phenotype and treatment selection. Paclitaxel's shorter survival outcomes likely indicate its use in patients with aggressive disease or visceral crisis. These findings underscore the variability in survival outcomes based on post-CDK4/6i therapy choices in HR+/HER2- MBC, with promising evidence for rechallenging strategies.

Conclusion: Switching CDK4/6i and ET conferred a statistically significant improvement of PFS in patients with progression or recurrence on prior CDK4/6i-containing therapy. These findings underscore the variability in survival outcomes based on post-CDK4/6i therapy choices in HR+/HER2- MBC, with promising evidence for rechallenging strategies.

## Introduction

About 70% of patients with metastatic breast cancer (MBC) are hormone receptor-positive (HR+) and human epidermal growth factor receptor 2-negative (HER2-). Cyclin-dependent kinase 4/6 inhibitors (CDK4/6i) in combination with endocrine therapy (ET), such as an aromatase inhibitor (AI) or fulvestrant, are the standard of care (SoC) for this subset of MBC tumors, in both first and second line, and in cases of endocrine resistance, in women independent of menopausal status, and in men [1-3]. After disease progression on CDK4/6i treatment, there is no standard of care on what to offer patients in the subsequent lines of systemic treatment. Several possibilities include the following: switching to another ET (in monotherapy), ET (tamoxifen, exemestane, or fulvestrant) with mammalian target of rapamycin inhibitor (mTORi) everolimus, a poly (ADP-ribose) polymerase (PARP) inhibitor (PARPi) such as talazoparib or olaparib for patients harboring a germline BReast CAncer gene (gBRCA) 1/2 mutation or a partner and localizer of BRCA2 (PALB2) mutation, the phosphoinositide 3-kinase (PI3K) inhibitors alpelisib and inavolisib for patients with somatic phosphatidylinositol-4,5-bisphosphate 3-kinase catalytic subunit alpha (PIK3CA) mutations, elacestrant (an oral selective estrogen receptor degrader {SERD}) with fulvestrant for patients with estrogen receptor alpha mutation (ESR1m), and capivasertib with fulvestrant for tumors exhibiting PIK3CA/serine-threonine kinase 1 (AKT1)/phosphatase and tensin homologue deleted on chromosome 10 (PTEN) alterations [4,5].

A new treatment option for this MBC subtype is antibody-drug conjugates (ADCs), namely, trastuzumab deruxtecan (T-DXd) [5]. Finally, there is the option of chemotherapy (ChT), the most common of which are capecitabine or paclitaxel. However, despite the growing number of available targeted and cytotoxic therapies, there is a lack of comparative real-world data or prospective studies evaluating how best to sequence these options, particularly in patients with more than one targetable alteration. The optimal treatment strategy after progression on CDK4/6i therefore remains uncertain. Trying to answer these questions and fill in this gap in the knowledge of MBC, we performed this real-world data analysis to evaluate what therapies were given after progression on a CDK4/6i. Our goal was to compare both progression-free survival (PFS) and overall survival (OS) on different therapies and try to find out if there is a sequencing that shows better outcomes.

A review of the evidence of HR+/HER- metastatic breast cancer treatment with CDK4/6i

A treatment based on ET is the preferred first-line option, even in the presence of visceral disease, with the exception of a visceral crisis. Chemotherapy (ChT) still is the first choice when treating patients with a visceral crisis [5]. Although the combination of CDK4/6i with ET is the standard of care, ET alone still has a role in the treatment of certain cases of MBC, such as low-volume disease, a long disease-free interval (DFI), patient preference, or the limited availability of CDK4/6i [5]. At present, the three CDK4/6i approved by the European Medicines Agency (EMA) and the US Food and Drug Administration (FDA) are palbociclib, ribociclib, and abemaciclib, and they have benefits in both OS and PFS, with no comparison head-to-head within a clinical trial [5]. The three drugs have different toxicity profiles as they have distinct inhibitory potencies. Although they share some characteristics of toxicity profile, such as neutropenia, CDK4/6i have some distinct individual features: Hematological toxicity is more common with palbociclib, ribociclib can cause hepatotoxicity and prolong QT interval, and abemaciclib is more associated with diarrhea [6].

PALOMA-2 with palbociclib, MONALEESA-2 with ribociclib, and MONARCH-3 with abemaciclib are the phase III trials that study CDK4/6i in the first line in postmenopausal women [7-10]. MONALEESA-7 only included pre- or peri-menopausal women and studied ribociclib with ET, in both first and second lines [11]. PALOMA-3 (palbociclib), MONALEESA-3 (ribociclib), and MONARCH-2 (abemaciclib) included patients progressing with a previous ET [12-15]. Palbociclib was the first CDK4/6i demonstrating benefit in PFS, in PALOMA-3 trial, when used in combination with fulvestrant versus fulvestrant alone, in patients progressing after ET, with a difference of 2.3 months (hazard ratio {HR}, 0.69; 95% confidence interval {CI}, 0.43-1.09) for endocrine resistance and 7.8 months (HR, 46; 95% CI, 0.36-0.59) for endocrine-sensitive population. Additionally, palbociclib also showed OS benefit in the second line (34.9 versus 28.0 months; stratified HR, 0.81; 95% CI, 0.64-1.03) [12]. In the second line, both ribociclib and abemaciclib have also presented benefits in PFS and OS. The combination of ribociclib with ET also demonstrated a significant benefit in terms of PFS (20.5 versus 12.8 months; HR, 0.59; 95% CI, 0.48-0.73) and OS (67.6 versus 51.8 months with ribociclib versus placebo; HR, 0.67; 95% CI, 0.50-0.90) in the MONALEESA-3 trial [13,14]. This is the longest median OS (67.6 months) observed for a first-line population in a phase III trial setting in MBC to date. The combination of abemaciclib with ET also showed a benefit in PFS (16.4 versus 9.3 months; HR, 0.55; 95% CI, 0.45-0.68) and OS (46.7 versus 37.3 months; HR, 0.76; 95% CI, 0.61-0.95) in the MONARCH-2 trial [15]. In the first line, palbociclib demonstrated statistically and clinically significant improvement in terms of PFS (24.8 versus 14.5 months; HR, 0.58; 95% CI, 0.46-0.72) but not OS, according to the last publications of the PALOMA-2 trial [7,16]. Abemaciclib also demonstrated an advantage in PFS (29.0 versus 14.8 months; HR, 0.54; 95% CI, 0.43-0.67) and OS (66.8 versus 53.7 months; HR, 0.80; 95% CI, 0.64-1.0), although the OS did not reach statistical significance in the MONARCH-3 trial [10]. In MONALEESA-2 and MONALEESA-7, ribociclib showed a PFS and OS benefit [8,11]. For postmenopausal patients treated in the first line, PFS was 25.3 versus 16 months (HR, 0.57; 95% CI, 0.46-0.70) and OS was 63.9 versus 51.4 months (HR, 0.76; 95% CI, 0.63-0.93), according to the latest results [8]. In the same way, pre- or peri-menopausal patients without previous treatment experienced this benefit in MONALEESA-7, with a median OS of 58.7 versus 48.0 (HR, 0.76; 95% CI, 0.61-0.96) [11].

In the clinic, AI are the selected ET in patients with de novo MBC or in those patients whose disease recurrence occurred more than 12 months after finishing adjuvant ET [17]. The PARSIFAL trial studied palbociclib combined with letrozole or fulvestrant, and fulvestrant was inferior to letrozole in terms of PFS, although with no statistical significance [18]. Therefore, fulvestrant should be the choice in those patients whose disease had progressed with AI or who had a recurrence of disease less than 12 months after finishing adjuvant ET [17,18]. A real-world study has demonstrated that first-line ChT or ET alone had worse median PFS when compared to the association of ET with CDK4/6i (22 months for combination therapy, 14 months for ET, and 12 months for ChT). In the first line, the combination therapy had a better improvement in PFS when compared to the second line (PFS1, 22 months, versus PFS2, 12 months) [3]. The previously mentioned trials did not categorize any factors that could predict more benefit from CDK4/6i, not even from one CDK4/6i over another [19].

Considering the final OS results, ribociclib is the CDK4/6i with the most consistent significant OS benefit across all phase III studies. Considering the patients with the presence of non-life-threatening visceral disease (lung, pleural, and/or liver disease), if we look at the main trials, we know that each study included up to 30%-40% of patients with these characteristics, and these performed similarly to other patients. The RIGHT Choice phase III trial randomly assigned 222 patients to receive ribociclib plus ET or combination ChT, with more than half (150, 67.6%) having symptomatic visceral metastases and 41 (18.5%) having rapid disease progression per investigator's judgment. The authors conclude that first-line ribociclib plus ET showed a significant PFS benefit, 21.8 months with ribociclib + ET (95% CI: 17.4-26.7) versus 12.8 months for the combination ChT arm (95% CI: 10.1-18.4), with a 0.61 HR (95% CI: 0.43-0.87). In addition, this demonstrated similar response rates (66.1% with ribociclib versus 61.8% in the ChT arm) and better tolerability over combination ChT in patients with clinically aggressive disease [20]. A more recent approach raises an important consideration about the sequencing of CDK4/6i. The phase III SONIA trial evaluated the efficacy, safety, and cost-effectiveness of CDK4/6i added to either first- or second-line ET [21]. This trial randomized 1050 patients and was meant to determine whether the first-line use of CDK4/6i combined with ET offers a significant PFS or OS advantage compared to second-line use. The median time on CDK4/6i was 24.64 months in first line and 8.08 months in the second line (Δ 16.56 months), with a median PFS2 of 31.0 versus 26.8 months, respectively. The trial found no statistically significant or clinically meaningful difference in PFS between the two sequencing strategies, showing that despite a prolonged use in the first line, this was associated with increased toxicity and higher costs. The authors suggest that reserving CDK4/6i for second-line therapy could be a viable approach for many patients [21]. The ABIGAIL trial is a randomized phase II study of abemaciclib plus ET with or without a short course of induction paclitaxel in patients with previously untreated HR+/HER2- MBC with aggressive disease criteria. The study confirms that first-line treatment with abemaciclib + ET leads to a higher early objective response rate (ORR) compared to ChT in patients who meet criteria for aggressive disease [22].

CDK4/6i post-progression treatments and outcomes

Since patients with luminal-like breast cancer can be treated with different therapeutic strategies, namely, ET, ChT, or targeted therapy, it was not expected to have a consensus on the subsequent line of treatment post-progression on CDK4/6i. Data from the phase III trials that led to the approval of ribociclib, palbociclib, and abemaciclib in first, second, or subsequent lines and data from the real world mirror this. In the five trials where CDK4/6i were given in the first line, on average, 65% of patients received ET as a subsequent therapeutic strategy, 44% received ChT, 18% received CDK4/6i, and 17% received mTOR inhibitors. After second-line CDK4/6i, namely, on MONARCH-2 and PALOMA-3 trials, ET alone was administered on average to 55% of patients, ChT in 66%, CDK4/6i in 9%, and mTOR inhibitors in 24% [23].

Two American real-world data studies report on the subsequent line of therapy used after CDK4/6i [1,24]. The first one was conducted between 2012 and 2017, only in postmenopausal women [24]. A post-progression therapy after CDK4/6i was identified in 35% of patients, of which only 39.6% has been treated with CDK4/6i in the first-line setting. Of patients treated with a first-line CDK4/6i strategy, 38% received ET in the second line, 35.6% ChT, 14.4% everolimus, 9.6% another CDK4/6i, and 2.4% other strategies. Of patients treated with second to fourth line with CDK4/6i, 33.8% received ET, 37.5% ChT, 2.6% everolimus, 15.1% another CDK4/6i, and 0.9% other strategies. This study also raises the hypothesis that rapidly progressing disease, metastatic site location, age, and ET partner may be predictive of subsequent regimen [24]. The other real-world data study reported specifically on the second-line therapy for 1210 patients treated with first-line CDK4/6i between 2015 and 2020. The majority of patients (88.2%) were treated with palbociclib with an endocrine partner in the first-line setting. Eight hundred thirty-nine patients were treated in the second line: ChT was the most common treatment administered (29.7%), followed by ET alone (12.4%). Everolimus was chosen for 11.7% of patients, and a minority of patients received alpelisib (1.9%) or PARPi (0.5%). Fifty-one patients (6.1%) were enrolled in a clinical trial [1,19].

There are many questions left to answer, and one of them is whether there is a role for the continuation of a CDK4/6i after progression on these drugs. This question is being addressed in clinical trials, including maintaining the same CDK4/6i and switching ET and others changing CDK4/6i [25]. The previously mentioned American study by Martin et al. (2022) that evaluated a population of 1210 patients with HR+/HER2- metastatic breast cancer showed that patients receiving palbociclib were more likely to maintain this therapy after progression (when compared to patients receiving ribociclib or abemaciclib), with 204 in 261 (78.2%) of the patients continuing with this CDK4/6i in the second-line setting. Also, ribociclib had a higher rate of patients that kept treatment with this CDK4/6i, specifically 14 out of 23 (60.9%) [1]. This study with retrospective data suggests that continuing with a CDK4/6i beyond the first progression (changing ET) may be an effective strategy, showing that these patients have better median real-world PFS (HR, 0.48; 95%, CI 0.43-0.53; p < 0.0001) and OS (HR, 0.30; 95% CI, 0.26-0.35; p<0.0001), when compared to patients receiving ChT [1].

A study published in 2021 that evaluated 30 women with HR+ MBC from a single institution addressed this question. The investigators reported data showing that maintaining treatment with CDK4/6i plus ET after initial progression was associated with a median PFS of 11.8 months (95% CI: 5.34-13.13 months). In this study, most of the patients received palbociclib + AI as initial therapy (67%), followed by palbociclib + fulvestrant (23%), palbociclib with other AI (20%), and abemaciclib with fulvestrant or letrozole (6%) [26]. Contrary to these findings, a study first presented at the 2022 San Antonio Breast Cancer Symposium, the PACE trial, advocates that the combination of palbociclib and fulvestrant did not prolong PFS compared to fulvestrant alone in the population of patients who had progressed with treatment with a CDK4/6i plus ET. This trial showed that a triplet combination of programmed death-ligand 1 (PD-L1) inhibitor avelumab + palbociclib + fulvestrant could prolong PFS but warrants further investigation in this patient population [27]. A multicenter, retrospective American study (2021) analyzed 87 patients with MBC from six medical centers receiving abemaciclib after prior CDK5/6i progression (palbociclib- or ribociclib-containing regimen) [27]. This study showed that when abemaciclib was used after prior CDK4/6i treatment, it was well tolerated and had a clinical benefit (at least a six-month duration of treatment) in 36.8% of patients. In this cohort, PFS and OS were similar to those observed in the MONARCH-1 study (5.3 months versus 6.0 months), in which none of the patients had received prior CDK4/6i therapy [28].

The MAINTAIN phase II multicenter, randomized trial was designed to evaluate the efficacy of exemestane or fulvestrant ± ribociclib in 119 patients whose cancer had previously progressed with CDK4/6i (any of them) + ET (any) [29]. There was a significantly improved PFS in patients who were treated with fulvestrant or exemestane + ribociclib, with a median PFS of 5.33 months (95% CI: 3.25-8.12 months) versus placebo (median PFS of 2.76 months; 95% CI, 2.66-3.25 months), with an HR of 0.56 (95% CI, 0.37-0.83; p = 0.004). For patients treated with fulvestrant, similar results were observed, with a median PFS for those receiving ribociclib of 5.29 months versus 2.76 months in patients receiving placebo (HR, 0.59; 95% CI, 0.38-0.91; p = 0.02). At 12 months, patients without progression were 25% on the ribociclib arm versus 7% on the placebo arm. The investigators concluded that switching ET and receiving ribociclib after progression on CDK4/6i significantly improves PFS [29]. The Post-MONARCH trial was the first phase III randomized, placebo-controlled study to show the benefit of continued CDK4/6 inhibition and switching the endocrine therapy beyond disease progression on a CDK4/6 inhibitor.

Abemaciclib + fulvestrant significantly improved PFS (six-month PFS rates of 50% and 37% in the abemaciclib + fulvestrant and placebo + fulvestrant arms) after disease progression on prior CDK4/6i + ET in patients with HR+/HER2- MBC, with a consistent treatment effect across different clinical and genomic subgroups, including patients with or without ESR1 or PIK3CA mutations [30]. The PALMIRA trial was a phase II trial that randomly assigned 198 patients to receive palbociclib plus second-line ET (letrozole or fulvestrant, based on prior ET) or second-line ET alone. This study showed that maintaining palbociclib with a second-line ET beyond progression on prior palbociclib-based therapy did not significantly improve PFS compared to second-line ET alone (median investigator-assessed PFS was 4.2 months {95% CI: 3.5-5.8} in the palbociclib + ET versus 3.6 months {95% CI: 2.7-4.2} in the ET arm {hazard ratio, 0.8; 95% CI, 0.6-1.1; p = 0.206}) [31,32]. In patients previously exposed to ET, ESR1m can emerge as a frequent cause of acquired resistance to the backbone therapy, particularly with AI [33]. The SERENA-2 phase II clinical trial assessed the efficacy of camizestrant versus fulvestrant. In this trial, only about 50% of patients received a CDK4/6i in the first line. This is a positive trial with a clinically meaningful benefit for camizestrant in terms of PFS [34]. Elacestrant is the first oral SERD whose efficacy was evaluated in a randomized, open-label, phase III trial (EMERALD trial) that included patients exposed to a previous line with a CDK4/6i and at least one line of ChT. ESR1 mutation was detected in 47.8% of patients, and patients were treated with elacestrant (n = 239) or standard of care (n = 238). The 12-month PFS rates in patients with ESR1 mutation was 26.8% (95% CI: 16.2-37.4) for elacestrant versus 8.2% for standard of care (95% CI: 1.3-15.1). This benefit, even though it was higher for the ESR1-mutated patients, was also observed in the overall population, with manageable safety [35].

The use of oral SERDs such as giredestrant and amcenestrant was also evaluated in phase II trials. No significant difference in median PFS in patients with ESR1 mutation with giredestrant (acelERA trial) or amcenestrant (AMEERA-3 trial) versus standard ET (in second line and beyond) [36,37]. Other options for treatment after CDK4/6i include exemestane + everolimus (EE), alpelisib for PIK3CA-mutated patients, capivasertib with fulvestrant for tumors exhibiting PIK3CA/AKT1/PTEN alterations, PARPi, and the new ADC agents [5]. A retrospective analysis studied the efficacy of EE in patients previously treated with ET + CDK4/6i. This analysis suggests that EE remains an effective treatment option for HR+/HER2- MBC following prior ET or ET + CDK4/6i, with median OS improvements primarily driven by prior CDK4/6i use rather than the timing of EE initiation [38]. The BYLieve phase II study, a small study performed in patients with prior exposure to CDK4/6i, showed the activity of alpelisib plus fulvestrant in patients with PIK3CA mutation, with a median PFS of 8.0 months (95% CI: 5.6-8.6) [39]. The CAPitello-291 phase III trial compared capivasertib + fulvestrant with fulvestrant alone. Median PFS in the overall and protein kinase B (AKT) pathway-altered population was 7.2 months (95% CI: 5.5-7.4) and 7.3 months (95% CI: 5.5-9.0) [40].

The recently presented and published EMBER-3 trial evaluated imlunestrant (an oral SERD) versus imlunestrant versus exemestane and fulvestrant. About 60% prior CDK4/6i treatment (nearly all palbociclib or ribociclib) and ESR1 mutations were found in 32%-42%. In patients with ESR1m, the PFS with imlunestrant versus SoC ET was 5.5 versus 3.8 months (HR, 0.62; 95% CI, 0.46-0.82), respectively. Imlunestrant + abemaciclib led to a 43% reduction in the risk of progression or death over imlunestrant alone in all patients. There was a consistent benefit of imlunestrant + abemaciclib across key clinical subgroups, regardless of ESR1m status [41]. The NCT03854903 is a phase I trial that tested a tyrosine kinase inhibitor (TKI), bosutinib, in combination with palbociclib and fulvestrant for HR+/HER2- MBC refractory to a CDK4/6i. The combination of bosutinib, palbociclib, and fulvestrant demonstrated a robust clinical benefit rate (CBR) of 50% and was well tolerated at the recommended dose, offering a promising strategy to overcome CDK4/6i resistance in HR+/HER2- MBC [42]. For patients with a germline BRCA mutation, PARPi such as olaparib and talazoparib are associated with an improved PFS and quality of life, but not OS, compared with single-agent ChT [43,44].

ADCs are now emerging as new treatment options in a variety of cancers. In patients with MBC with HER2-low expression (a score of 1+ or 2+ on immunohistochemical {IHC} analysis and negative results on in situ hybridization {ISH}) or ultra-low HER2 expression (IHC of 0 with membrane staining) who had received one or more lines of endocrine-based therapy and no previous chemotherapy for metastatic breast cancer, trastuzumab deruxtecan (T-DXd) was compared to ChT in the DESTINY-Breast06 trial. This treatment showed higher response rates and a prolonged PFS [45]. Sacituzumab govitecan (SG), an antibody-drug conjugate targeting trophoblast cell-surface antigen 2 (TROP-2), which is upregulated in breast cancer, received approval in endocrine-resistant HR+/HER2- MBC, with a median OS improvement of 3.2 months on single agent ChT [46]. Treatment with datopotamab deruxtecan (Dato-DXd) generated a statistically significant and clinically meaningful improvement in PFS, meeting the study's co-primary endpoint of the phase III TROPION-Breast01 trial. Patients treated with Dato-DXd (n = 365) achieved a median PFS of 6.9 months (95% CI: 5.7-7.4) versus 4.9 months (95% CI: 4.2-5.5) for those given investigator's choice of ChT (n = 367), translating to a 37% reduction in the risk of disease progression or death (HR, 0.63; 95% CI, 0.52-0.76; P < 0.0001). Unfortunately, this trial did not significantly improve OS compared to the investigator's choice of ChT, according to findings from the final OS analysis published by the company [47].

## Materials and methods

We conducted a multicentric retrospective observational study in 10 Portuguese centers. Patients who had received at least one CDK4/6i in combination with ET in the metastatic setting, in both first line and subsequent treatment lines, between January 2016 and July 2022, were selected. Inclusion criteria were as follows: having a histologically confirmed diagnosis of HR+/HER2- (HER2 0, 1, or 2+ with negative ISH) breast cancer and having metastatic disease at diagnosis or a recurrence of localized disease at the time of the analysis; eligible patients were at least 18 years old and have been treated with at least one CDK4/6 inhibitor in metastatic setting (first or subsequent line) between January 2016 and July 2022. All patients had experienced disease progression with a CDK4/6i. Exclusion criteria included the following: other active tumors, whether undergoing treatment or not. Data were collected from medical records by the team of each hospital included. We collected data on the date of birth, the date of diagnosis of breast cancer, initial staging (stages I-IV), HR status (progesterone and estrogen), Ki67, HER2 status (0, 1+, or 2+ with negative ISH), the date of recurrence (if recurrence occurred), the number of metastatic sites, and the location of metastasis. Regarding the treatment, we collected the following data: the type of treatment for metastatic disease (from first to fourth line), the date of the beginning and end of each treatment, the date of progression, and the motive for interrupting treatment (toxicity, progression, and others). There was no limit on the number of prior ChT regimens/cycles, ET, or targeted therapies allowed. For data analysis and quality assurance, all collected data up to December 2023 were systematically processed, validated for accuracy, and analyzed using appropriate statistical methods to ensure reliability and consistency.

The primary objective of this study was to compare PFS to OS across different systemic treatment options administered as second or later lines, after progression on CDK4/6 inhibitors in patients with HR+/HER2- metastatic breast cancer.

The secondary objectives were to identify the post-CDK4/6 inhibitor treatment associated with the most favorable clinical outcomes and evaluate the prognostic impact of metastatic pattern, specifically comparing outcomes between patients with bone-only disease and those with extra-osseous metastases.

We define PFS as the time between the initiation of therapy and clinician-recorded progression or death. OS was defined as the time between the diagnosis and death or the last confirmed visit with the patient alive. Survival curves were estimated with the Kaplan-Meier method, and the survival of different populations was compared with the pairwise log-rank test at a significance level of 0.05. Due to the exploratory nature of our work, we did not correct for multiple comparisons. To analyze the effect of bone and visceral metastases on PFS and OS, we used a bivariate Cox proportional hazards model, using the presence of bone metastases and the presence of extra-osseous metastases as binary variables. Again, we chose a significance level of 0.05. Descriptive statistics, survival analysis, and data visualizations were performed with a custom script written in the Python programming language (Python Software Foundation, Fredericksburg, VA) using the following software packages: Pandas (for data manipulation and descriptive statistics), Lifelines (for survival analysis), and Matplotlib (for data visualization and plotting) [48-51]. The study was approved by the Ethics Committee of Hospital Professor Doutor Fernando Fonseca (approval number 017/2025), ensuring compliance with ethical guidelines for research involving human participants.

## Results

We identified 237 patients with MBC who were treated with at least one CDK4/6i. Data from the patient characteristics, such as Eastern Cooperative Oncology Group (ECOG) Performance Status (PS) at the diagnosis and initial staging of disease, are listed in Table 1. The mean age was 54.7 ± 14.1 years at the time of metastatic disease diagnosis, with 155 (65.4%) patients having a World Health Organization (WHO) PS of 0 at diagnosis. Most of the patients were post-menopausal at diagnosis (144, 60.8%). In our population, the majority of patients were not diagnosed with a stage IV disease (67.6%). The majority had visceral disease (182 patients, 76.8%). Table 2 refers to the tumor characteristics in the metastatic setting. Among the 237 patients analyzed, the mean number of metastatic sites was 1.76 ± 0.96. A single metastatic site was observed in 51.9% of cases (n = 123), while 48.1% (n = 114) had multiple metastatic sites. Regarding metastatic patterns, 23.2% (n = 55) had bone-only metastases, 24.5% (n = 58) had visceral-only metastases, and the majority (52.3%, n = 124) presented with both bone and visceral metastases (Table 2).

**Table 1:** Patient characteristics Patient characteristics for the study population, namely, age, ECOG performance status, hormone status at diagnosis, and tumor stage at the time of diagnosis ECOG, Eastern Cooperative Oncology Group; SD, standard deviation

	N = 237	
Age (years, mean ± SD)	54.7 ± 14.1	
Sex		
Female	235	(99.2%)
Male	2	(0.8%)
ECOG Performance Status score, number (%)		
0	155	(65.4%)
1	73	(30.8%)
2	8	(3.4%)
3	1	(0.4%)
Hormonal status at diagnosis, number (%)		
Pre-menopausal	81	(34.0%)
Peri-menopausal	10	(4.2%)
Post-menopausal	144	(60.8%)
Not applicable (male patients)	2	(0.8%)
Initial staging, number (%)		
I	33	(13.9%)
II	49	(20.7%)
III	71	(29.9%)
IV	77	(32.4%)
Unknown	7	(2.9%)

**Table 2:** Tumor characteristics in metastatic setting SD: standard deviation

	(N = 237)	
Number of metastatic sites		
Mean (SD)	1.76 ± 0.96	
Single site versus multiple site metastasis, number (%)		
Singe site	123	(51.9%)
Multiple sites	114	(48.1%)
Metastatic pattern, number (%)		
Bone only	55	(23.2%)
Visceral only	58	(24.5%)
Bone and visceral	124	(52.3%)

In the Cox proportional hazards model, the presence of bone metastases was a predictive factor for worse PFS in a multivariate analysis (HR, 1.49; 95% CI, 1.03-2.15; p = 0.03). Extra-osseous metastases were not predictive of either better or worse PFS. No type of metastases (bone or extra-osseous) was predictive of better or worse OS (Table 3).

**Table 3:** Cox proportional hazards model for the prediction of survival as a function of metastatic pattern This table shows the coefficients for the Cox proportional hazards model using the presence of bone metastases and extra-osseous disease as predictors of progression-free survival (PFS) and overall survival (OS). The top three rows refer to the coefficients for the PFS prediction, and the bottom three lines show the coefficients. The p value for the presence of bone metastases as a predictor of PFS is <0.05, which is statistically significant. The presence of extra-osseous disease is not a statistically significant predictor of PFS. No metastatic pattern was predictive of overall survival CI, confidence interval; HR, hazard ratio

	HR	95% CI	P value
Progression-free survival			
Bone metastases	1.49	1.03-2.15	0.03
Extra-osseous disease	1.32	0.91-1.92	0.15
Overall survival			
Bone metastases	1.32	0.87-1.98	0.19
Extra-osseous disease	1.51	0.98-2.31	0.06

Information from treatments in the metastatic setting is summarized in Table 4. A total of 189 patients (79.7%) received a CDK4/6i in combination with ET as a first-line treatment, 49 (20.6%) as a second line, and 12 (7.0%) in third line. After progression with CDK4/6i, the next line of treatment included ET alone in 67 patients (28.3%), capecitabine in 67 (28.3%) patients, paclitaxel in 30 patients (13.5%), a rechallenge strategy with a different CDK4/6i in 14 patients (5.4%), other ChT in 21 patients (8.9%), and other treatments in 39 patients (16.4%).

**Table 4:** Treatments performed in metastatic disease Systemic treatments have been grouped by the line of treatment (first line, second line, and third line) and by category. Categories were defined according to the mechanism of action CDK4/6i: cyclin-dependent kinase 4/6 inhibitors

	N (%)	
First line	N = 237	
CDK4/6i	189	(79.7%)
Ribociclib	87	(36.7%)
Palbociclib	80	(33.8%)
Abemaciclib	22	(9.3%)
Endocrine therapy without CDK4/6i	26	(11.0%)
Chemotherapy	18	(9.3%)
Second line	N = 237	
CDK4/6i	49	(20.6%)
Ribociclib	16	(6.7%)
Palbociclib	23	(9.7%)
Abemaciclib	10	(4.2%)
Endocrine therapy without CDK4/6i	66	(27.4%)
Chemotherapy	91	(38.4%)
Others	32	(13.5%)
Third line	N = 172	
CDK4/6i	12	(7.0%)
Ribociclib	6	(3.5%)
Palbociclib	5	(2.9%)
Abemaciclib	1	(0.6%)
Endocrine therapy without CDK4/6i	30	(17.4%)
Chemotherapy	109	(63.7%)
Others	20	(11.7%)

Table 5 demonstrates the benefit of each treatment option after CDK4/6i, showing a higher OS benefit for patients who receive ET or who are rechallenged with a different CDK4/6i. The data highlight notable differences in PFS and OS across treatment modalities. ET and capecitabine, both used in 28.3% of patients, showed relatively similar PFS, but ET had a longer median OS (19.8 versus 14.5 months). Paclitaxel demonstrated the shortest PFS (4.9 months) and OS (9.4 months), suggesting limited long-term efficacy in this cohort. Interestingly, patients who underwent rechallenge with a different CDK4/6 inhibitor had the longest PFS (10.1 months), though the median OS was not reached, indicating potential benefit in selected cases. Non-taxane/non-capecitabine chemotherapy showed highly variable outcomes, with wide confidence intervals suggesting heterogeneity in response. These findings emphasize the need for personalized treatment strategies based on disease characteristics and prior therapies.

**Table 5:** Outcomes with treatments after CDK4/6i This table compares the PFS and OS according to the systemic treatment after the CDK4/6 inhibitor. Less common forms of chemotherapy were grouped under the label non-taxane/non-capecitabine chemotherapy CDK4/6i, cyclin-dependent kinase 4/6 inhibitors; ChT, chemotherapy; CI, confidence interval; NR, not reached; OS, overall survival; PFS, progression-free survival

Treatment	N (%)	Median PFS (months)	95% CI for PFS	Median OS (months)	95% CI for OS
Endocrine therapy	67 (28.3%)	6.9	5.1-8.6	19.8	14.0-26.0
Capecitabine	67 (28.3%)	8.4	6.7-17.5	14.5	10.4-19.9
Paclitaxel	30 (13.5%)	4.9	3.2-7.5	9.4	5.0-14.8
Rechallenge with a different CDK4/6i	14 (5.4%)	10.1	8.7-38.9	NR	18.2 to NR
Non-taxane/non-capecitabine ChT	21 (8.9%)	8.5	0.7-21.5	8.5	0.7-21.5

Table 6 shows the results of the pairwise log-rank p tests for statistical significance. For significance testing for overall survival comparisons of subsequent lines after treatment with a CDK4/6 inhibitor (Table 6), we tested the rechallenge with a different CDK4/6 inhibitor, hormone therapy (without the concomitant use of a CDK4/6 inhibitor), and paclitaxel or non-taxane chemotherapy. Other rare lines of treatments, such as the use of mTOR inhibitors or tailored targeted treatment, were not included because of low numbers. Comparisons with statistically significant differences (p < 0.05) are highlighted in bold. In particular, rechallenge with a CDK4/6i was shown to be better than capecitabine, paclitaxel, and non-taxane ChT. ET without a concomitant CDK4/6i was shown to be superior to capecitabine, paclitaxel, and non-taxane ChT. Comparisons between other options did not show statistically significant differences.

**Table 6:** Comparisons of PFS for subsequent lines after treatment with a CDK4/6 inhibitor This table shows the results for the log-rank p test used to determine whether PFS for one line of treatment is significantly longer or shorter. Comparisons were considered to be significant if the p value was <0.05 (in bold text) CI, confidence interval; PFS, progression-free survival; CDKi, cyclin-dependent kinase inhibitor; CDK4/6, cyclin-dependent kinase 4/6

		P value
CDKi	Capecitabine	0.01
	Hormone therapy	0.10
	Non-taxane chemotherapy	<0.005
	Paclitaxel	<0.0005
Capecitabine	Hormone therapy	0.01
	Non-taxane chemotherapy	0.09
	Paclitaxel	0.14
Endocrine therapy	Non-taxane chemotherapy	<0.005
	Paclitaxel	<0.005
Non-taxane chemotherapy	Paclitaxel	0.67

**Figure 1:**
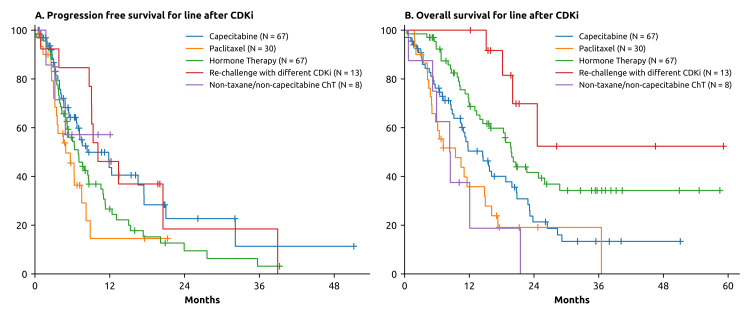
Progression-free survival and overall survival according to subsequent line after progression under a CDK4/6 inhibitor Kaplan-Meier curves have been drawn for each sub-population. Vertical markers represent censored data CDKi, cyclin-dependent kinase inhibitor; ChT, chemotherapy; CDK4/6, cyclin-dependent kinase 4/6

**Figure 2:**
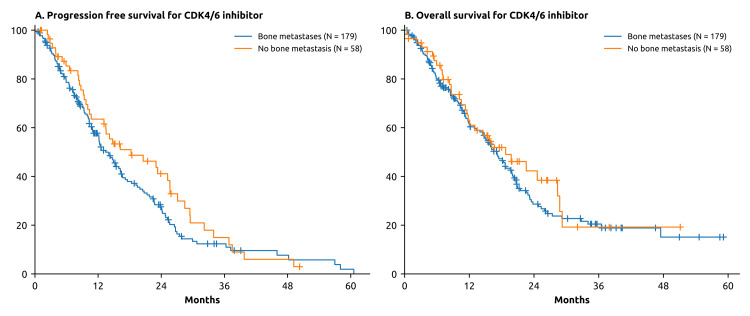
Progression-free survival and overall survival according to the presence or absence of bone metastasis Kaplan-Meier curves have been drawn for each sub-population. Vertical markers represent censored data CDK4/6: cyclin-dependent kinase 4/6

Figure 1 and Figure 2 represent the outcomes according to the subsequent line after progression on a CDK4/6 inhibitor and the presence or absence of bone metastases, respectively.

## Discussion

Multiple lines of hormone-based therapy are the standard of care in patients with HR+/HER2- MBC, although resistance almost invariably emerges. ﻿﻿The three CDK4/6i in combination with ET seem to be consistent and comparable in prolonging PFS in the metastatic setting, with differences in OS and toxicities [7,8,10,11,13]. According to the available data, rechallenge with a CDK4/6i (ribociclib) in patients pretreated and resistant to palbociclib needs confirmation and should only be considered in very selected patients (MAINTAIN trial) [29]. ﻿﻿New SERDs are in development, with elacestrant showing a significant benefit and being an EMA-approved option in ESR1 mutation tumors, mainly in patients exposed to CDK4/6i therapy for more than one year [35]. Alpha-selective PIK3CA inhibition emerged as an option for PIK3CA mutation tumors in combination with fulvestrant (or letrozole) [39]. ﻿﻿BRCA-mutated tumors benefited from PARPi [43,44]. ADCs emerged as a therapeutic option in HR+, as well as in low-expression HER2 in later-line therapy (SG, Dato-DXd, and T-DXd) [45-47].

The potential benefits of rechallenging with a different CDK4/6i are shown in our study, with this group achieving the highest OS (not reached). Although this strategy was only used in 5.4% of patients, it demonstrated a survival advantage that warrants further investigation, especially given the limitations of CDK4/6i trials in addressing optimal sequencing. This may also reflect a selection bias within the study, since patients chosen for ET (versus ChT) are likely to have less aggressive disease phenotypes and a more favorable prognosis. The relatively favorable PFS and OS observed with capecitabine (8.4 months and 14.5 months) suggest that it may serve as a viable option for disease control. However, the OS was higher in patients treated with ET alone (19.8 months). Paclitaxel, despite being a commonly used ChT, showed a notably shorter PFS and OS (4.9 months and 9.4 months, respectively). This likely reflects its use in patients with more aggressive disease, possibly those experiencing visceral crisis, where immediate cytotoxic effects are prioritized over longer-term survival.

Our study has some limitations inherent to a retrospective multicentric study, including potential variability in data collection across centers and the challenges associated with missing or incomplete data.

## Conclusions

Our study highlights the variability in survival outcomes based on the choice of therapy following CDK4/6i progression in patients with HR+/HER2- MBC. Notably, rechallenging with a different CDK4/6i may provide an OS advantage, supporting the emerging evidence. ET post-progression offered a relatively favorable OS, emphasizing its role in patients with less aggressive disease phenotypes. Capecitabine emerged as a viable option for disease control, while paclitaxel appeared more suited to patients with aggressive disease presentations, reflecting its use in managing visceral crises. Because treatment assignment was not randomized but instead chosen by the attending oncologist according to the patient's characteristics, favorable OS comparisons may reflect differences in disease characteristics more than the effect of different treatments. Thus, while our data are informative, it needs to be complemented with randomized prospective trials. This review and multicentric retrospective study underscores the complexity of treatment sequencing in HR+/HER2- MBC following progression on CDK4/6i, highlighting the importance of tailoring treatment strategies to individual patient profiles, considering factors such as disease biology, prior responses, and metastatic patterns. Future research should focus on optimizing treatment sequences and identifying predictive biomarkers to guide therapy selection, ultimately improving outcomes for patients with HR+/HER2- MBC.
